# Can they imagine the future? A qualitative study exploring the skills employers seek in pharmaceutical sciences doctoral graduates

**DOI:** 10.1371/journal.pone.0222422

**Published:** 2019-09-09

**Authors:** Jacqueline E. McLaughlin, Lana M. Minshew, Daniel Gonzalez, Kelsey Lamb, Nicholas J. Klus, Jeffrey Aubé, Wendy Cox, Kim L. R. Brouwer

**Affiliations:** 1 Center for Innovative Pharmacy Education and Research, Division of Practice Advancement and Clinical Education, University of North Carolina at Chapel Hill Eshelman School of Pharmacy, Chapel Hill, North Carolina, United States of America; 2 Division of Pharmacotherapy and Experimental Therapeutics, University of North Carolina at Chapel Hill Eshelman School of Pharmacy, Chapel Hill, North Carolina, United States of America; 3 Center for Integrative Chemical Biology and Drug Discovery, Division of Chemical Biology and Medicinal Chemistry University of North Carolina at Chapel Hill Eshelman School of Pharmacy, Chapel Hill, North Carolina, United States of America; 4 Division of Chemical Biology and Medicinal Chemistry, University of North Carolina at Chapel Hill Eshelman School of Pharmacy, Chapel Hill, North Carolina, United States of America; 5 Division of Practice Advancement and Clinical Education, University of North Carolina at Chapel Hill Eshelman School of Pharmacy, Chapel Hill, North Carolina, United States of America; Universidade de Brasilia, BRAZIL

## Abstract

Concerns about the extent to which graduate programs adequately prepare students for the workplace have prompted numerous calls for reform. Understanding what employers look for in doctoral graduates can help schools better align graduate training with workplace needs. Twelve pharmaceutical scientists across diverse specialties and career pathways described the skills considered requisite for success in today’s science economy. Depth and breadth of knowledge, communication, collaboration, adaptability, research productivity, experiential training, and motivation and drive were among the themes identified. These results can be used to inform the development of doctoral curricula in the biomedical sciences.

## Introduction

Career pathways in the pharmaceutical sciences are increasingly diverse, in part due to changing workforce needs and evolving student interests. In academia, for example, the number of biomedical doctorate recipients has increased significantly as the number of available tenure-track academic positions has declined [1, 2]. There is approximately one tenure-track position in the United States for every 6.3 Doctor of Philosophy (PhD) graduates, which means that more than 80% of new biomedical graduates are pursuing careers outside of academia [3]. Furthermore, graduate interest in tenure-track faculty positions often drops over the course of a PhD program [4]. As a result, more graduates are now pursuing alternative career pathways such as jobs in the private sector at established and startup pharmaceutical, biotechnology or biomedical research-related companies, research institutes, government regulatory agencies, non-profit organizations and non-research focused positions [4, 5].

The evolution of career pathways is further influenced by recent and rapid advancements in science. New technologies, techniques, materials, processes, and regulations can influence what types of jobs are available and what skills are needed in the workforce. In the pharmaceutical sciences, for example, rapid growth in the application of mathematical modeling and simulation in drug discovery and development has significantly increased the demand for well-trained and experienced quantitative scientists [6]. The application of artificial intelligence at all stages of drug discovery and development—from data mining and assistance in target identification and validation, to lead compound selection and prediction of drug candidate properties and risks–requires knowledgeable, “next generation” pharmaceutical scientists. Advances in gene editing, immuno-oncology, the role of the microbiome in disease pathogenesis and treatment, and precision medicine necessitate a scientific workforce with broad training in the chemical, biological and translational sciences in addition to scientific expertise in specific areas of the pharmaceutical sciences. In addition, there is a need for scientists who compare the effectiveness of available medications used to treat the same condition; evaluate implementation of new interventions and approaches to improve access to effective medications; and conduct pharmaceutical policy analyses.

Despite these changes, graduate training in the biomedical sciences remains largely unchanged. Doctoral students in the United States typically spend approximately 1 to 2 years taking coursework and complete 3 to 5 years of research under the close guidance of a faculty member. While digital age advancements are evident, such as improved access to published information and on-demand training, these advances have had little impact on the basic structure of graduate training and mentoring. The student-faculty apprenticeship model relies heavily on the knowledge, perspective, and quality of faculty mentors, and results in widely variable training experiences for students. Since many faculty have limited experience in careers outside of academia [7], they may be ill-equipped to provide guidance about non-academic careers [8] and subsequently encourage students to pursue tenure-track academic careers [4]. This disconnect can result in a significant gap in the support provided to, and the development of, those students who are considering non-academic positions [9].

Concerns about the extent to which graduate programs adequately prepare scientists for the workplace have prompted numerous calls for reform. Doctoral graduates often struggle to align their highly specialized knowledge and skills with the needs of employers while companies criticize the shortage of adequately prepared prospects [10]. Although a growing number of advocates propose rethinking doctoral training in an effort to better prepare graduates for success [1, 9, 11, 12, 13], few studies clearly identify the specific skills required by graduates in today’s rapidly evolving pharmaceutical industries. In the pharmaceutical sciences, most draw from faculty perspectives or literature reviews to identify core skills for graduate training. Wu Pong and colleagues [14], for example, synthesized literature to identify skills needed in the pharmaceutical industry, which included flexibility, creativity, leadership, communication, proposal writing, mentoring, business skills, and collaboration. More recently, the 2016–2017 Research and Graduate Affairs Committee of the American Association of Colleges of Pharmacy arrived at six domains of core competencies for graduate training in the pharmaceutical sciences: foundational knowledge, research, scientific communication, education, leadership and management, and personal and professional development [15].

Amid recent economic, professional, and technical changes, more research is necessary to better understand what skills graduates need to be competitive on the job market and advance the pharmaceutical sciences [14]. Inquiring directly with employers to explore how graduate programs can better support the development of key skills and abilities in graduates is an important step for aligning graduate training with workplace needs. The goal was to identify core skills that employers look for when hiring doctoral graduates from the pharmaceutical sciences.

## Methods

In March, 2017, the UNC Eshelman School of Pharmacy charged a committee with re-envisioning graduate education in the pharmaceutical sciences, which would provide the foundation for transformation of our pharmaceutical sciences graduate program. The committee consisted of 10 faculty members representing each of the School’s five divisions, two doctoral students, a staff member from the Office of Curricular and Student Affairs, and a health sciences librarian. As part of its charge to rethink the doctoral curricula at the School, the committee decided to interview experts across various sectors of pharmaceutical sciences in order to better understand the contemporary landscape of the pharmaceutical sciences discipline and the skills graduates needed to be successful within that landscape.

### Participants

Interviews were conducted in November and December 2017 with 12 identified experts representing expertise across all five academic divisions at the School: Chemical Biology and Medicinal Chemistry; Pharmacoengineering and Molecular Pharmaceutics; Pharmacotherapy and Experimental Therapeutics; Practice Advancement and Clinical Education; and Pharmaceutical Outcomes and Policy. At least 2 experts were identified and recommended by members of each academic division, ensuring that responses reflected the various areas of the pharmaceutical sciences. Eight experts were external to the School and four held positions within academia. In addition, pharmaceutical scientists with diverse expertise, career paths and current positions were interviewed, including global pharmaceutical companies, small start-up companies, government, and academia. Each participant was contacted via email and invited to participate in a 60-minute interview. Twelve out of 14 invited experts agreed to participate (85.7% response rate).

### Data collection

Each expert received information about the purpose of the interview, along with the interview questions prior to the interview. Interviews were conducted either in person or synchronously via Zoom [San Jose, CA] depending on the expert’s availability. All interviews were recorded and transcribed verbatim for analysis purposes. All members of the committee were invited to attend the interviews. Interviews were semi-scripted, with three primary areas of inquiry: changes in drug discovery, development and evaluation and its effect on graduate student training; important graduate student attributes; and ideas for graduate training. Additional probing questions were asked by committee members, as needed, to clarify or facilitate further discussion with the expert [See Appendix].

### Data analysis

The analysis focused on a single research question: What do graduates of our Pharmaceutical Sciences PhD programs need to be able to do/demonstrate to be successful? Grounded theory shaped the coding and analysis process [16]; six members of the committee volunteered to serve as data analysts and were responsible for open coding four of the 12 interviews for attributes, skills, and abilities described by the experts. Data analysts were assigned interview transcripts that were not in their area of expertise, and each interview transcript had at least two coders from different academic divisions. Each person was provided a guiding document and instructions on how to open code an interview transcript. After reading through the interview transcript, analysts were asked to note important statements that focused on the skills future graduates from the pharmaceutical sciences would need. Next, analysts were instructed to create a code descriptor along with an example quote from the transcript to illustrate their code.

Once all transcripts were open coded, one committee member (JM) compiled the initial coding from all transcripts to synthesize the data and identify emerging themes. Seven themes emerged through the initial review process. These themes were presented along with relevant quotes from the interviews to the research team for review; the team discussed any discrepancies or disagreements. Following this consensus building, results also were shared with School faculty during a retreat for questions and feedback. An independent qualitative analysis expert (LM) coded the transcripts following these processes. The inter-rater reliability for the thematic analysis coding was 0.99 with a 95% confidence interval, suggesting that there was excellent inter-rater reliability between the coders. These qualitative research steps (i.e., multiple coders, peer-debriefing, member-checking) promote trustworthiness of the findings.

### Ethical considerations, consent

This project was submitted to the UNC Institutional Review Board and processed under case number 18–0275. The submission was reviewed by the Office of Human Research Ethics, which determined it did not constitute human subjects research as defined under United States federal regulations [45CFR 46.102 (d or f) and 21 CFR 56.102(c)(e)(l)] and did not require IRB approval. Although consent was not required, all interviewees were informed of the purpose of the interviews (i.e. program evaluation and improvement, aimed at informing the revision of the School’s graduate program) during the recruitment phase and again at the start of the interview and all agreed before starting the interview.

## Results

The thematic analysis revealed seven broad themes about the skills and abilities employers look for when hiring pharmaceutical sciences doctoral graduates. Four of the themes were expressed by all 12 experts: knowledge, communication, collaboration, and motivation and curiosity. Three themes were salient, yet not present in every interview: adaptability, experiential training, as well as critical thinking and problem solving. In addition, ideas provided by participants for better aligning graduate training programs with workforce needs were compiled. Descriptions of each theme are presented below and in Table 1, along with relevant example quotes. While the overarching themes emerged as distinct from one another, they were not always mutually exclusive, and some quotes occasionally overlapped multiple themes. For example, some comments about communication overlapped with collaboration since communication is an inherent aspect of working with others.

**Table 1:** Themes and summary descriptions of skills employers seek in pharmaceutical science doctoral graduates.

Theme	Summary
**Knowledge**	• Demonstrate depth of knowledge in a specific area of pharmaceutical sciences • Exhibit breadth of generally applicable pharmaceutical sciences knowledge • Possess strong research and methodological skills
**Communication**	• Display effective verbal and written communication skills • Show ability to communicate effectively within an interdisciplinary setting
**Collaboration**	• Work productively with others in an interdisciplinary setting
**Motivation & Curiosity**	• Embody characteristics of internal motivation • Exemplify excitement and curiosity for research • Utilize self-directed learning to acquire new knowledge and skills
**Adaptability**	• Develop and enhance skills as the field changes • Pursue life-long learning • Be flexible and resilient
**Experiential Training**	• Acquire real work experiences during graduate school • Gain experience in a non-academic setting prior to graduation
**Critical Thinking & Problem Solving**	• Demonstrate ability to think through a complex problem • Describe problem solving processes to others • Possess strong analysis skills • Know when to seek help in solving a complex problem

### Knowledge

Every expert identified knowledge, in some capacity, as critical in prospective employees. The experts discussed knowledge in terms of depth and breadth, research achievements, and an individual’s familiarity with new and emerging methods. One expert stated, “*Above everything*, *I look for people who have good*, *good*, *solid*, *scientific training*, *background*, *and understanding… they’re going to have to really show*, *at least for their area*, *[that] they have depth of knowledge*.” Another expert echoed this sentiment by stating, “*there is an aspect of depth*, *so the assumption is you have depth in some things and if you look at those that are successful they have a deep understanding*.”

While depth was considered necessary, it was not considered sufficient. Experts wanted individuals who also had a breadth of knowledge across various areas, demonstrating their intellectual diversity. For example, one expert stated, “*we’re really looking for diversity of thought*,” while another stated, “*I like to have it both ways in the sense that we do like people to bring in an area of specialization*, *that may be cutting edge*, *may help to push us to the vanguard of an area*, *but our work in general is very broad*.*”*

In addition to depth and breadth of knowledge, experts discussed knowledge related to research. Six of the experts specifically referenced scholarly publications as an important achievement and indicator of knowledge and knowledge-based ability. For instance, one expert stated, “*I would also say publications are pretty important …it’s unfortunate*, *sort of superficial in a way*, *but we do definitely look for those [individuals] that have very high-profile publications*.” Experts indicated that the number and type of publications, along with the journals they are published in are held in high regard when considering a recent graduate for employment.

Finally, graduate students’ knowledge about new and emerging methods was considered high priority. Areas mentioned during interviews included bio-informatics, chem-informatics, translational sciences, and the use of big data and data science. Experts also emphasized that the field of pharmaceutical sciences is changing. One expert said, “*I feel like we’re entering another phase of the information age*, *so really big data and data science it’s an emerging field*. *We’re flooded with many sources of data and to be able to analyze that data*, *that involves computer and software skills*.*”* Still another expert identified other methodological changes that need attention for chemistry-oriented applicants when they said, “*You don’t see a lot of total synthesis anymore*, *so we look more at methodology*. *If I’m looking at somebody to do chemical biology work*, *we would look more for somebody perhaps with more of a mix of a little bit of synthetic-organic chemistry*, *but a bit more biochemistry and some experience that could apply to the chemical biology work*.*”* Here the experts acknowledged that the industry is changing and pharmaceutical sciences graduates must be equipped with knowledge that is contemporary and relevant to emerging areas of science.

### Communication

Experts indicated that graduates need to be able to communicate in verbal and written form while also demonstrating the ability to listen. Many experts indicated that “*being able to translate your work*” so that a variety of people could understand it was essential. Experts noted the increasingly interdisciplinary nature of the pharmaceutical sciences and the expectation that scientists communicate effectively and efficiently across various contexts and audiences. One expert noted, “*there’s a different level of communication*, *when you are talking to another scientist versus when you’re talking to a patient or you’re talking to a clinician*.” More specifically, “*the ability to really hone down on what’s important and present it [the research] in a way so that someone who is intelligent but not a subject area expert could understand and really understand the impact*.” Said more simply, “*Can you listen to people and can you get your point across*?*”*

Although verbal communication was a major focus, experts also identified strong written communication as a necessity. As noted by one participant, “*I see abstracts from individuals who I know are very smart*, *but I have no idea what they’re trying to communicate to me*.” Several experts also noted deficits in written communication, with one expert stating, “*well*, *writing is a lost art*” and another, “*I see more challenges with written communication than oral*” indicating that strong written communication could be a skill that differentiates new graduates.

### Collaboration

Comments about communication often were coupled with those emphasizing collaboration. Every expert expressed the need for pharmaceutical sciences graduates to possess the ability to collaborate with others, using terms such as teamwork, groups, multidisciplinary, fit, shared goals, and cross-divisional. One expert stated, “*the first thing that I look for is for people who can work in teams*, *very important in the industry*.” Discussions regarding collaboration indicated that graduate students needed to demonstrate their ability to work productively with others, whether those individuals were in their lab setting or in an interdisciplinary setting. One expert noted looking for graduates that “*understand how they fit within that team*, *when it’s appropriate for them to speak up*, *the type of information for them to communicate…”*

### Motivation and curiosity

This theme is unique in that every expert mentioned this element, yet described it using different terms and descriptions. Some called it “curiosity,” “innovation,” “that extra little spark,” “driven,” or “ability to grow professionally.” Despite these variations, all experts spoke to an innate element of an individual that set them apart from others. The experts were looking for individuals who would thrive under pressure, be able to anticipate change, and create or identify opportunity. One expert described it as they “*come with a different mindset*” while another noted, “*we are looking for people that are forward thinking…that will take on responsibility to educate themselves*.*”* Another expert expressed, “*I look for fire in the belly*. *Are they excited*? *Can they imagine the future*?” Although, the experts described this quality in different ways, all descriptions reflected upon an individual’s potential to be successful in a variety of capacities within the workplace.

### Adaptability

Experts identified adaptability as another important trait for graduate students to possess, with one expert noting, “*the nature of the drug discovery business is always changing*, *so you just have to hire smart people who are adaptable*, *who are open to learning*, *learning new skills*.” This statement suggests that graduates cannot remain stagnant with the skills they obtain during their graduate studies. To remain competitive, they will need to keep pace with the rapid growth and changes in the pharmaceutical sciences. Additionally, the experts expressed that graduates need to convey a desire to grow, be “*flexible and resilient”*, and welcome challenges in their work.

### Experiential training

Experts suggested that graduates with experience in more “*real-world application*” and stronger networks within the pharmaceutical sciences were often more competitive applicants. As noted by one, “*It’s not totally where you come from and what you know*, *it’s who you know*.*”* Another acknowledged real world experience as nearly universal in their new hires, “*A lot*, *if not all*, *of the individuals we bring in…have had some background doing a project within the industry*.*”* Several suggested that pharmaceutical sciences programs should implement “*short-term rotations…to just get a sense of what [industry is] like*,” which would “*allow the individual to come in*, *get an understanding of how the industry works*, *and get an understanding of what kind of science is being done and how that person can integrate themselves into that type of science*.” This suggests that experiential training during graduate school would be beneficial for both the student and the future employer.

### Critical thinking and problem solving

The final theme focused on the ability of graduate students to work through difficult problems. Experts indicated that graduate students must be able to work through problems on their own while also knowing when to seek help. As one expert stated, “*what I’m really looking for is how do people approach the problem that they had*? *What did they contribute to solving that problem*? *How they went about solving the problem*, *and what they delivered at the end*.*”* Relatedly, experts highlighted strong research skills as a core component of problem solving and agreed that the ability to demonstrate strong analysis skills can set graduates apart from other applicants. As noted by one expert, “*Once they’ve done an experiment or analysis*, *how do they put the information together to really link it back to the question that they were supposed to be addressing in the beginning*?”

### Improving graduate training

Participants provided numerous suggestions for improving graduate training, aimed at better preparing graduates for success in the workplace and aligning graduate programs with the needs of the workforce. Engaging students in more situated learning with hands-on experience was a common suggestion. As suggested by participants: “*the last three years need to be 90%*, *or more*, *hands-on in the lab research;*”

“*build in team-based projects where everybody has to pull their weight and figure out how to interface to achieve a common goal*;” and “*allow the graduate student to spend some time with either a research lab or our organization*.*”* In addition, participants encouraged the integration of more training in communication: “*Teaching individuals how to write a good abstract is a very good exercise*.” and “*if [graduates] are going to have to work with other people who don't have their background then I think it's very important to learn to work with and communicate [with] them while you're in a PhD program*.*”* Lastly, several participants noted that while health care has changed, “*health care will continue to dramatically change*, *based on finances*, *based on the economy*, *based on a lot of different factors*”, highlighting the need for ongoing evaluation and revision of graduate training programs to ensure that they remain contemporary and relevant.

## Discussion

Graduate programs must equip students with the skills most valued and needed by the marketplace [14, 17]. This is a unique challenge in today’s science economy amid technological advancements, diversifying career pathways, and shifting student interests. As doctoral graduates oversaturate the academic job market [1, 2], graduate programs must position students to develop skills that both differentiate them and meet workforce needs.

This work provides insight into what employers seek in doctoral graduates within the rapidly evolving world of the pharmaceutical sciences. Our results align closely with literature describing skills considered necessary for success across other industries, such as Tony Wagner’s New World of Work and the Seven Survival Skills [18]. Wagner identified seven core skills: critical thinking and problem solving, collaboration across networks and leading by influence; agility and adaptability; effective oral and written communication; initiative and entrepreneurialism; accessing and analyzing information; and curiosity and imagination [18]. More specifically, our results align with literature reviews and faculty commentaries in the pharmaceutical sciences concerning core skills, such as foundational knowledge, flexibility, creativity, communication, collaboration, and research [14, 15]. Our experts’ emphasis on breadth of knowledge, experiential training, and research productivity may help graduate programs better understand needs of the job market and, subsequently, the needs of students preparing for that market.

Identifying strategies that optimize student development is imperative for graduate programs aimed at differentiating students. As stated by Wagner, “the most successful companies in the emerging economy need a new and very different kind of worker who teams with others to continuously reinvent the machine as well as the products and services it creates (pg. 41)” [18]. How can graduate programs help students develop skills and abilities that differentiate them in a competitive job market? Furthermore, how can programs achieve this aim for multiple, diverse career pathways within a single curriculum? Our findings suggest that discrete specialization in an area of the pharmaceutical sciences is essential, but no longer sufficient. In addition to depth, graduates need breadth of knowledge, along with the ability to communicate and collaborate across broad audiences and contexts, as pharmaceutical sciences becomes more interdisciplinary.

In addition, lab rotations and experiential training in diverse settings such as industrial or regulatory sectors could provide critical, real-world experience. Our experts stressed a desire for graduate students to have broad professional experiences and exposure to other programs prior to graduation. This might require graduate programs to engage in collaborative communities, not only within and among schools of pharmacy, but through partnerships with industry and government agencies. The Cooperative Research Centres (CRC), for example, have reconceptualized the role and style of doctoral education to support the needs of students amid changing industry needs by providing them with access to training opportunities in a variety of sectors [19, 20]. Students enrolled in the CRC doctoral program often feel better equipped to handle the communication and collaborative demands of jobs in the industrial sector after their workplace experience [19]. As another example, the Centers of Excellence in Regulatory Science and Innovation (CERSI) program provides a formal mechanism to establish collaborations between the U.S. Food and Drug Administration (FDA) and academic institutions (e.g., UCSF-Stanford, Johns-Hopkins). A key expectation is that these centers provide training and professional development opportunities focused on regulatory science, with the help of FDA staff [21].

Further, programs should consider strategies for preparing graduate students for various career pathways, including those outside of academia [9]. Since faculty mentors may not be prepared to provide broad career guidance for jobs outside of academia [7, 22], training and infrastructure may be necessary to support this approach. The NIH Director’s Biomedical Research Workforce Innovation Award: Broadening Experiences in Scientific Training (BEST) (DP7), for example, was created to support institutions whose faculty and staff were working to design and establish new models for training and development programs for biomedical doctoral students [22]. The “Preparing Future Professions” at the University of Kentucky also aims to fill the gap between graduate training and nonacademic career aspirations [23].

An individual’s choice to pursue graduate education in the biomedical sciences can be described by human capital theory [24]. This traditional economic perspective assumes that individuals make decisions by weighing the benefits against the costs for all possible alternatives, and then selecting the best alternative [25, 26]. Benefits for students may include improved likelihood of securing employment that is aligned with their intellectual as well as financial interests. For employers, a graduate degree can signify that the prospective employee is capable of performing the job in question [27, 28, 29], principally improving his or her human capital. However, if our graduate programs fail to equip students with the knowledge and skills needed and valued by employers, the capital associated with a doctoral degree could deteriorate, prompting employers to devalue graduate training.

Calls for reform in the biomedical sciences highlight apparent disconnects between graduate programs and the job market. Advocates have proposed broadening doctoral curricula, for example, to better prepare biomedical PhDs for a wide range of science-related career paths [1, 9, 11, 12]. Others have suggested that the biomedical PhD be restructured into two degrees, one degree geared for academia and a second to train those who would like in-depth science knowledge for use in other careers [11]. Additional recommendations have included the use of master’s degree programs for industry-seeking candidates, limiting the number of individuals who are allowed to pursue a PhD [1, 11, 12], reshaping doctoral training to focus more on critical thinking and scientific rigor [30], and revising current curricula to include more collaborative, personalized, and interdisciplinary work [31]. While most advocate for curricular change, Callier and Vanderford [32] recommend empowering PhD trainees to better understand and navigate the priorities of the academic institutions, stating that “trainees cannot depend on the university to prepare them for their careers; instead, they must take the initiative to research possibilities, create opportunities, and build a career on their own terms.”

Amid these calls for graduate education transformation, this work is a first step toward better understanding what our graduate program must do to better prepare students for the new era of drug discovery and development. Given the importance of this issue, we were surprised by the dearth of research to support evidence-based decision making in graduate education and curriculum design. Additional research is clearly needed to inform the development of graduate curricula that align with market needs, optimize student learning, and promote advancements in science. Exploring the types of activities and strategies used by graduate students to successfully prepare for the job market, for example, could uncover programs, activities, courses, or technologies that could strengthen curricula. Better understanding the needs of faculty mentors as they help prepare graduate students for increasingly diverse employment opportunities could also be critical for positioning our graduate programs for success.

### Limitations

There are several limitations to note. First, there was a relatively small sample size. Second, the experts were identified by faculty from a single, yet scientifically-diverse, school. Third, this was limited to experts from the pharmaceutical sciences. While these limitations can constrain generalizability, the methods provided a rich depth of information and promoted trustworthiness of findings. Clear and consistent themes emerged across the interviews, representing expert viewpoints from various career pathways and diverse specializations within the pharmaceutical sciences, which could also be considered relevant for various areas of related science.

## Conclusions

The employability of doctoral students is a key concern to educators and employers. To attract and support students in today’s competitive market, doctoral programs must demonstrate to applicants that they will have the qualities and skills most valued by future employers when they graduate [17]. We interviewed experts from various career pathways and pharmaceutical sciences specializations to identify the skills employers seek in prospective employees. Depth and breadth of knowledge, communication, collaboration, and motivation are among the most desirable qualities in the pharmaceutical sciences. These findings have important implications for developing doctoral programs in the pharmaceutical sciences, as well as other biomedical sciences.

## Appendix

### Interview questions

Changes in Drug Discovery, Development, and Evaluation

1 How do you see drug discovery, development and evaluation changing in the next 5–10 years? How does this affect the type of training/skillset that graduate students in pharmaceutical sciences will need to have?
2 What do you see as “cutting edge” areas in the pharmaceutical sciences that graduate programs should consider integrating into their training?

Graduate Attributes

3 In your experience, what skills does an exemplary graduate of a pharmaceutical sciences program possess?
4 What are common deficiencies that you encounter in new graduates with degrees in pharmaceutical sciences?
5 What are the most important attributes you look for in hiring, in order, and with relative weight specified?

Graduate Training

6 What innovative approaches can graduate training programs use to help graduate students develop “soft skills”?
7 Can you think of innovative ways that pharmaceutical companies and/or regulatory agencies can collaborate with academia for graduate training?
